# Comparative evaluation of rumen metagenome community using qPCR and MG-RAST

**DOI:** 10.1186/2191-0855-3-55

**Published:** 2013-09-11

**Authors:** Neelam M Nathani, Amrutlal K Patel, Prakash S Dhamannapatil, Ramesh K Kothari, Krishna M Singh, Chaitanya G Joshi

**Affiliations:** 1 Department of Animal Biotechnology, College of Veterinary Science & Animal Husbandry, Anand Agricultural University, Anand, Gujarat 388 001, India; 2 Department of Microbiology, Christ College, Vidhya Niketan, P.B. No.05, Rajkot-5, Gujarat, India; 3 Department of Biosciences, Saurashtra University, Rajkot-5, Gujarat, India

**Keywords:** Real time PCR, MG-RAST, *Bubalus bubalis*, *Fibrobacter succinogens*, *Ruminococcus flavefaciens*, *Prevotella bryantii*, *Selenomonas ruminantium*, *Methanomicrobiales*

## Abstract

Microbial profiling of metagenome communities have been studied extensively using MG-RAST and other related metagenome annotation databases. Although, database based taxonomic profiling provides snapshots of the metagenome architecture, their reliability needs to be validated through more accurate methods. Here, we performed qPCR based absolute quantitation of selected rumen microbes in the liquid and solid fraction of the rumen fluid of river buffalo adapted to varying proportion of concentrate to green or dry roughages and compared with the MG-RAST based annotation of the metagenomes sequences of 16S r-DNA amplicons and high throughput shotgun sequencing. Animals were adapted to roughage-to-concentrate ratio in the proportion of 50:50, 75:25 and 100:00, respectively for six weeks. At the end of each treatment, rumen fluid was collected at 3 h post feeding. qPCR revealed that the relative abundance of *Prevotella bryantii* was higher, followed by the two cellulolytic bacteria *Fibrobacter succinogens* and *Ruminococcus flavefaciens* that accounted up to 1.33% and 0.78% of the total rumen bacteria, respectively. While, *Selenomonas ruminantium* and archaea Methanomicrobiales were lower in microbial population in the rumen of buffalo. There was no statistically significant difference between the enumerations shown by qPCR and analysis of the shotgun sequencing data by MG-RAST except for *Prevotella.* These results indicate the variations in abundance of different microbial species in buffalo rumen under varied feeding regimes as well as in different fractions of rumen liquor, i.e. solid and the liquid. The results also present the reliability of shotgun sequencing to describe metagenome and analysis/annotation by MG-RAST.

## Introduction

Culture dependent methods for enumerating bacterial abundance are known to be biased since it is possible to cultivate bacteria only if their metabolic and physiological requirements can be fulfilled *in vitro*. Fluorescence-based methods like the flow cytometry can also be used to enumerate bacteria (Veal et al. 2000). However, most bacteria are observed to be optically similar and thus require artificial modification of the target bacteria using fluorescent labeling (Attfield et al. 1999). Differences in bacterial size and the presence of different contaminating matrices (e.g. feed particles, soil) may also interfere in microscopic enumeration (Veal et al 2000). Rapid enumeration of bacteria is also possible using a variety of molecular approaches (Hugenholtz et al. 1998; von Wintzingerode et al. 1997).

Molecular methods have shown that the complexity of microbial communities is much greater and that the majority of rumen microbes are still unknown (Leser et al. 2002; Pryde et al. 1999) due to the failure of many bacteria to grow in a given culture medium (Huijsdens et al. 2002; Langendijk et al. 1995). Quantitative molecular methods could be more sensitive and selective than traditional methods as they do not rely on the ability of bacteria to grow. Primers with broad interspecies specificity have been designed to amplify 16S rDNA by PCR and have been used to determine bacterial abundance in complex communities (Klausegger et al. 1999; Marchesi et al. 1998; Suzuki et al. 2000). qPCR, such as the SYBR green system, allows rapid detection and quantification of DNA (Heid et al. 1996). In addition, the in-built 96-well format greatly increases the number of samples that can be simultaneously analyzed. Conserved regions of 16S rDNA provide the means for detecting and enumerating complex bacterial populations by qPCR. The final determination of bacterial load by real-time PCR in a multi-species population may be influenced by the variation in the number of rRNA operons in a given species (Farrelly et al. 1995). However, in a variety of complex environmental, industrial and health-related situations in which multi-species populations are sampled along with impurities, other methodologies are likely to be far less sensitive or precise. This information is thus applied to determine the anaerobic bacteria load in the community samples. Moreover, DNA-based methods offer the option of storing samples until their analysis, which could be an important advantage.

The complex symbiotic microbiota of the rumen is responsible for breakdown of plant fiber, an ability the ruminal host animal lacks. This microbiota is highly responsive to changes in diet, age, antibiotic use, and the health of the host animal and varies according to geographical location, season, and feeding regimen (Bryant 1959; Tajima et al. 2001). Earlier rumen microbiology used cultivation-based techniques for monitoring such changes, but recent developments of more accurate molecular detection methods have brought new improvements in the field (Tajima et al. 2001). The availability of isolates representing the main groups of general diversity offers clear advantages with the possibility of assigning the functional role in the rumen based on the metabolic properties of pure cultures. Detection probes or PCR primers for rumen bacteria have been used for detection and quantification of the corresponding species in the rumen by qPCR approach. High complexity of rumen microflora has led researchers to find particular microbial groups that could serve as an indicator of health promoting microbiota. Studies have consistently reported a high prevalence of anaerobic bacteria within the complex rumen microbial community.

Recently, random community genomics, or metagenomics, where DNA is sequenced directly from environmental samples has provided insights into microbial communities. DNA is sequenced without cloning, using next-generation sequencing techniques. Regardless of the sequencing approach used, first step in analysis of any metagenome involves comparing the sequences to known sequence databases. Subsequent analysis including phylogenetic comparisons, functional annotations, binning of sequences, phylogenomic profiling, and metabolic reconstructions need to be computed. MG-RAST server is a freely available, fully automated open source system for processing metagenome sequence data based on the SEED framework for comparative genomics (McNeil et al. 2007; Overbeek et al. 2005). The server provides several methods to analyze the different data types, including phylogenetics and the ability to compare one or more metagenomes.

The objective of this work was to determine the bacterial quantification in rumen samples and to compare the qPCR results with those obtained by using MG-RAST server for metagenomic analysis. We hypothesized that bacteria, including total bacteria, and anaerobic bacteria, could be reproducibly quantified from rumen samples using qPCR, and it would have comparable sensitivity and specificity to MG-RAST predicted quantities. We chose *Ruminococcus flavefaciens, Fibrobacter succinogens, Prevotella bryantii, Selenomonas ruminantium* and methanomicrobiales targets as these are the most common microorganisms present in the rumen microflora. The panel of anaerobic bacteria was chosen based on metagenomic shotgun sequencing data from our laboratory as they are frequently present in buffalo rumen metagenome samples.

## Materials and methods

### Sample collection

The experiment for metagenomic analysis of ruminal microbes was carried out on 8 Mehsani buffalo reared at Livestock Research Station, Sardarkrushinagar Dantiwada Agricultural University, Gujarat. Four animals were fed on 50:50, 75:25 and 100:00 dry roughage to concentrate ratio. Another four animals of the same group were fed on 50:50, 75:25 and 100:00 green roughage to concentrate ratio over uniform feeding regime of six weeks. Samples were collected on the last day (42 days dietary period), 3 hours post feeding using flexible stomach tube and vacuum pump from rumen of the buffalo. The rumen fluid was then passed through muslin cloth, without pressure for removal of particulate matter, and the filtrate aliquoted as fractions of 200 μl in cryo vials was stored at −80°C for further study. About 200 mg of solid fraction, after removing liquid by squeezing, was aliquoted in sterile tubes and then immediately frozen into liquid nitrogen (Additional file 1: Online Resource 1).

### DNA extraction and PCR amplification of 16S RNA genes

Total DNA was extracted separately by using commercially available QIAmp DNA stool mini kit (Qiagen, USA). The total DNA mixture was used as a template in PCR to amplify 16S rDNA. Species specific PCR primers were used for the amplification of target region of the 16S rRNA genes. The target DNA of total bacteria, *Fibrobacter succinogens, Prevotella bryantii, Ruminococcus flavefaciens, Selenomonas ruminantium* and archaea were amplified from the metagenomic DNA, as described previously (Koike and Kobayashi 2001; Muyzer et al. 1993; Tajima et al. 2001).

### Preparation of DNA standard for absolute quantification by qPCR

Plasmid DNA containing the respective target DNA fragment was used as DNA standard in qPCR. The target DNA was amplified using the species-specific primer sets (Table 1). After confirmation of the expected size amplification on an agarose gel, the PCR products were excised from the gel, purified using the QIAquick gel extraction kit (Qiagen, CA), and then ligated into pTZ57RT cloning vector (Fermentas, UK). The ligated products were transformed to competent *Escherichia coli* DH5 alpha cells by heat shock. Plasmids were purified from positive clones using a QIAprep spin miniprep kit (Qiagen, CA), and cloned in pTZ57RT cloning vector using InsTAclone PCR cloning kit (Fermentas, UK). The recombinants were screened by colony PCR with respective primer sets and plasmids from positive clones were purified using a QIAprep spin miniprep kit. The concentration of the plasmid was determined with a Nanodrop spectrophotometer (Thermoscientific). Copy number of each standard plasmid was calculated using formula; Copy No/μl = Concentration of plasmids (gm/μl) × 6.022 x 10^23^ / length of recombinant plasmid (bp) × 660, (660 = Molecular weight of one basepair, 6.022 × 10^23^ = Avogadro’s number). Ten-fold dilution series ranging from 10^9^ to 10^2^ copies were prepared for each target. Quantitative PCR was performed with ABI 7500 FAST real time PCR system (Applied Biosystems, USA) using QuantiFast SYBR green PCR mastermix (Qiagen, CA). The amplification reactions were performed in a total volume of 15 μL, containing 30 ng of template DNA, 7.5 μL of 2X SYBR green master mix, 0.5 μL of each primer (10 pmol /μL) and sterile H_2_O. The cycling conditions consisted of initial denaturation step at 95°C for 5 min, followed by 40 cycles of 95°C 15 s and 60°C for 1 min. The 10-fold dilution series of the standard plasmid for the respective target was run along with the corresponding samples in triplicate. Fluorescence was measured once every cycle after the extension step using filters for SYBR Green (excitation at 492 nm and emission at 530 nm). The normalized fluorescence data were converted to a log scale and the threshold was determined to calculate the threshold cycle value (*Ct*; the cycle at which the threshold line crosses the amplification curve). Upon completion of real-time PCR run, data were automatically analyzed for melt curve and quantification by 7500 system Sequence Detection Software (SDS).

**Table 1:** PCR primers for detection of rumen bacteria

**Target bacterium**	**Primer**		**Product**
	**Forward primer**	**Reverse primer**	**Size(bp)**
Total bacteria	CCTACGGGAGGCAGCAG	ATTACCGCCGCTGTTGG	194
*Prevotella bryantii*	ACTGCAGCGCGAACTGTCAGA	ACCTTACGGTGGCAGTGTCTC	540
*Fibrobacter succinogenes*	GGTATGGGATGAGCTTGC	GCCTGCCCCTGAACTATC	445
*Ruminococcus flavefaciens*	GGACGATAATTGACGGTACTT	GCAATCYGAACTGGGACAAT	520
*Selenomonas ruminantium*	TGCTAATACCGAATGTTG	TCCTGCATCAAGAAAGA	513
*Methanomicrobiales*	ATCGRTACGGGTTGTGGG	CACCTAACGCRCATHGTTTAC	506

### Shotgun sequencing

The DNA samples were subjected to sequencing based on ion semiconductor technology using Ion Torrent PGM as per the manufacturer’s instructions. The steps in brief included enzymatic fragmentation of DNA to obtain fragments in the range of 280 to 300 bp size. The desired size fragments were ligated with the library adaptors and subjected to emulsion PCR, followed by its recovery and loading onto Ion Torrent PGM 316 chip.

### 16 s r-DNA amplicon pyrosequencing

Specific hypervariable regions in the 16 s rDNA were targeted for amplicon sequencing. The fusion primers were designed so as to include the priming site for sequencing primer, MIDs, and target specific sequences. DNA from liquid and solid fractions was used as template to generate amplicons of targeted hypervariable region. Four sets of primers viz. 8F and 534R, 517F and 926R, 917F and 1386R and 1099F and 1541R were used to generate amplicons. The amplification was performed using the emulsion PCR master mix (Roche diagnostics, USA) in the presence of 3% DMSO. The expected size amplicons were gel purified using QIAquick gel extraction kit. The gel purified amplicons were quantified by Nanodrop 1000 spectrophotometer. The number of molecules of the amplicons was estimated by molar conversion formula. The amplicons were diluted to 10^9^ molecules per μl. Equal amount of amplicons from four sets of primers (i.e. 8F & 534R, 517F & 926R, 917F & 1386R and 1099F & 1541R) for each sample (i.e. Rumen Liquid DNA and Rumen Solid DNA) were pooled and further diluted to 10^7^ molecules/μl concentration. The amplicon library was then subjected to emulsion PCR using the GS-FLX Titanium kit (Roche diagnostics, USA). The recovery and enrichment of the beads and pyrosequencing was carried out as described in the protocol.

### Data analysis

The sequence data was processed by 454 run processing software to remove the adaptor sequences and to filter out the low quality sequence reads. The reads corresponding to each amplicon were separated using the Perl script based on MIDs. The high quality reads for each of the samples were analyzed by MG-RAST for taxonomic profiling of the metagenomic data.

### Statistical analysis

One way ANOVA was carried out using SPSS (Statistical Package for Social Sciences) to check the statistical significance of the results. Spearmen’s correlation test was performed to determine the strength of relationships between the qPCR and MG-RAST analysis for samples sequenced using shotgun (shotgun sequencing/MG-RAST) and amplicon sequencing (amplicon sequencing/MG-RAST), respectively. After performing the statistical analysis using the proportions of bacterial species obtained for all the 48 samples, the mean and standard deviations of green and dry liquid as well as solid samples of four animals were calculated for interpretation of results.

## Results

### Real time PCR

C_T_ values decreased at least three fold on each dilution between 10^9^ and 10^2^ molecules/μl which were used as standards. In the reaction for all standard, nearly perfect linear regressions, intercept and slope were obtained between threshold cycle and quantities of standard for all targets and data generated from the reaction were used for further analysis.

### Enumeration of 4 bacterial species; an archaea and total bacterial population

Rumen fluid of buffalo, fed on varied concentrations of green and dry roughage, and fractionated into liquid and solid, were compared for bacterial abundance by qPCR and the results were compared with the MG-RAST based analysis of the same samples sequenced using shotgun and amplicon sequencing methods. The total bacteria in the rumen were estimated to be >10^6^ (detailed values in Additional file 2: Online Resource 2). Statistical analysis showed that the proportion of bacterial species detected by qPCR was quite similar to that shown by shotgun sequencing, while amplicon sequencing showed significant variation compared to the qPCR results except for *Ruminococcus flavefaciens*. A positive correlation was observed between qPCR and shotgun sequencing (Spearman rank correlation; r = 0.772, p < 0.01) whereas negative correlation was seen between PCR and amplicon sequencing analysis (Spearman rank correlation; r = −0.382, p < 0.01). Majority of samples processed by amplicon sequencing showed much higher proportions of the bacterial species studied in the present study in comparison to the other methods used for quantification (viz. qPCR and shotgun sequencing). On performing One-way ANOVA it was found that the variation between the results (in percentage) of real time PCR and shotgun sequencing was insignificant for all bacterial species except for the *Prevotella bryantii* population.

**Figure 1:**
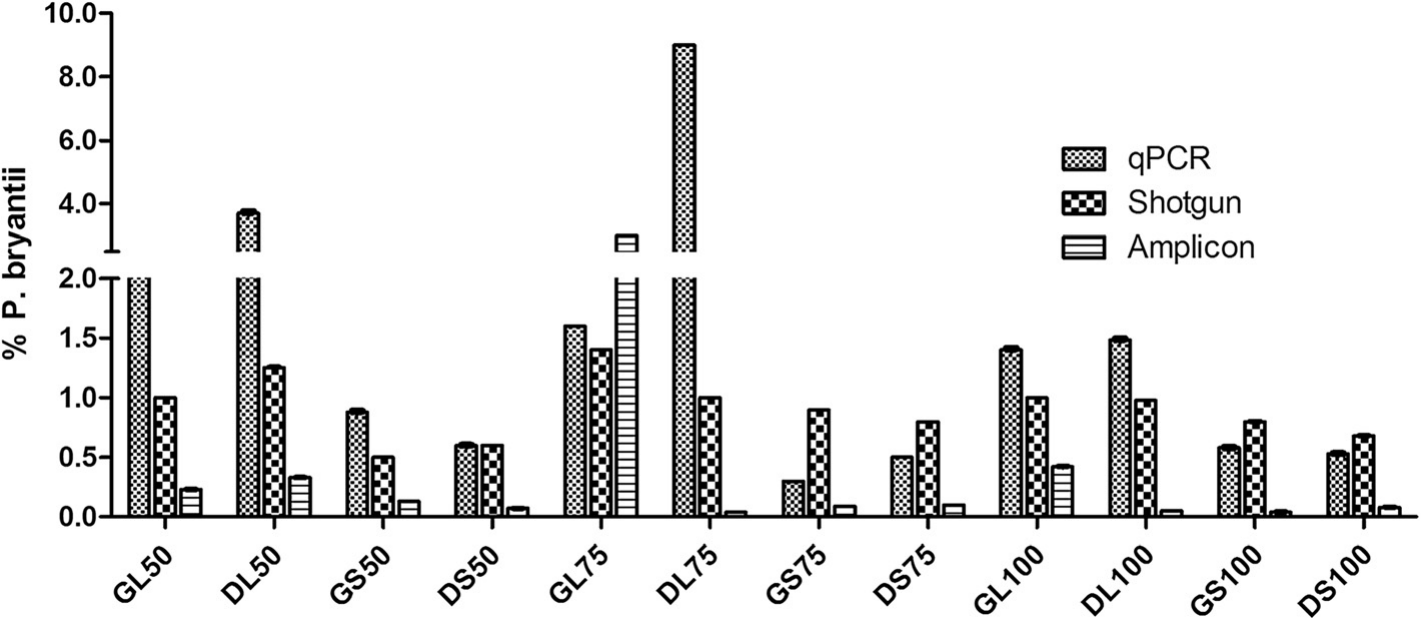
***P. bryantii *proportion as shown by qPCR and MG-RAST based analysis of shotgun and amplicon sequencing data.** GL50, 75 and 100: Rumen Liquid fraction from 50%, 75% and 100% green roughage fed animals, respectively. GS50, 75 and 100: Rumen Solid fraction from 50%, 75% and 100% green roughage fed animals, respectively. DL50, 75 and 100: Rumen Liquid fraction from 50%, 75% and 100% Dry roughage fed animals, respectively. DS50, 75 and 100: Rumen Solid fraction from 50%, 75% and 100% Dry roughage fed animals, respectively.

For *Fibrobacter succinogens,* the proportion observed was slightly lesser by quantification using qPCR as compared to the values obtained by the MG-RAST analysis of the shotgun sequences (Table 2). In spite of the difference in values, *Fibrobacter* abundance was more in the solid fraction of fluid in animals fed on green roughage while in animals fed on dry roughage it was more in the liquid fraction of the fluid. One of the other major cellulolytic bacteria usually found in the rumen samples, *Ruminococcus flavefaciens,* was observed to be more in the solid and liquid fraction of the rumen samples in animals fed with dry roughage compared to green roughage (Table 3). While the *Prevotella bryantii* species showed dominant prevalence among the four bacterial species quantified by us, and was in higher proportion in the liquid fraction of both green and dry fed animals of all three roughage combinations as shown by qPCR and MG-RAST results (Figure 1). Comparative abundance analysis of the two cellulolytic bacterial species *F. succinogens* and *R. flavefaciens* obtained by qPCR with the taxonomic distribution as predicted by MG-RAST on the basis of ribosomal RNA genes annotation showed ambiguous results (Additional file 3: Online Resource 3 and Additional file 4: Online Resource 4).

**Table 2:** **Comparison of *Fibrobacter succinogens *percentage by qPCR and MG-RAST analysis for same samples processed by shotgun and amplicon sequencing**

**Sample**		***Fibrobacter succinogens *(% ± SD)**		
		**qPCR**	**Shotgun**	**Amplicon**
50% roughage	GL	0.33 ± 0.005	0.90 ± 0.001	0.33 ± 0.002
	DL	0.35 ± 0.001	0.70 ± 0.000	0.70 ± 0.004
	GS	0.20 ± 0.001	0.43 ± 0.0005	0.35 ± 0.001
	DS	0.11 ± 0.0007	0.38 ± 0.0005	0.37 ± 0.001
75% roughage	GL	0.40 ± 0.003	1.23 ± 0.005	3.00 ± 0.000
	DL	0.38 ± 0.003	1.63 ± 0.011	1.75 ± 0.009
	GS	0.10 ± 0.0004	0.50 ± 0.0008	0.70 ± 0.000
	DS	0.02 ± 0.000	0.40 ± 0.0008	0.28 ± 0.002
100% roughage	GL	0.20 ± 0.002	0.53 ± 0.001	0.55 ± 0.311
	DL	0.78 ± 0.003	2.00 ± 0.000	3.00 ± 0.000
	GS	0.06 ± 0.0005	0.38 ± 0.002	0.18 ± 0.112
	DS	0.01 ± 0.000	0.28 ± 0.0005	0.09 ± 0.020

*GL* = Green liquid, *DL* = Dry liquid, *GS* = Green solid, *DS* = Dry solid.

**Table 3:** **Comparison of *Ruminococcus flavefaciens *percentage by real-time PCR and MG-RAST analysis for same samples processed by shotgun and amplicon sequencing**

		***Ruminococcus flavefaciens *(% ± SD)**		
**Sample**		**qPCR**	**Shotgun**	**Amplicon**
50% roughage	GL	0.45 ± 0.002	0.18 ± 0.0009	0.35 ± 0.002
	DL	0.45 ± 0.002	0.10 ± 0.000	0.30 ± 0.0008
	GS	1.33 ± 0.006	0.50 ± 0.0008	0.98 ± 0.0005
	DS	0.58 ± 0.004	0.53 ± 0.0009	1.07 ± 0.008
75% roughage	GL	0.40 ± 0.002	0.20 ± 0.000	0.30 ± 0.000
	DL	0.48 ± 0.001	0.23 ± 0.0005	0.40 ± 0.000
	GS	0.55 ± 0.004	0.35 ± 0.001	1.00 ± 0.000
	DS	0.77 ± 0.004	0.38 ± 0.0005	1.00 ± 0.000
100% roughage	GL	0.20 ± 0.010	0.15 ± 0.0005	0.35 ± 0.001
	DL	0.25 ± 0.002	0.25 ± 0.0005	0.75 ± 0.001
	GS	0.24 ± 0.0008	0.30 ± 0.000	0.85 ± 0.008
	DS	0.63 ± 0.005	0.60 ± 0.0008	1.33 ± 0.005

*GL* = Green liquid, *DL* = Dry liquid, *GS* = Green solid, *DS* = Dry solid.

We also quantified the firmicutes *Selenomonas ruminantium* bacteria and archaea methanomicrobiales in the buffalo rumen samples and the percentage of both were in accordance with those predicted by the shotgun sequencing/MG-RAST (Tables 4 and 5). These results showed that the annotations by MG-RAST were showing much higher abundance of these species as compared to the values obtained from qPCR results.

**Table 4:** **Comparison of *Selenomonas ruminantium *percentage by qPCR and MG-RAST analysis for same samples processed by shotgun and amplicon sequencing**

		***Selenomonas ruminantium *(% ± SD)**		
**Sample**		**qPCR**	**Shotgun**	**Amplicon**
50% roughage	GL	0.063 ± 0.0003	0.003 ± 0.000	0.400 ± 0.003
	DL	0.040 ± 0.0001	0.005 ± 0.000	0.430 ± 0.001
	GS	0.006 ± 0.000	0.004 ± 0.000	0.750 ± 0.002
	DS	0.008 ± 0.000	0.003 ± 0.000	1.000 ± 0.000
75% roughage	GL	0.090 ± 0.000	0.004 ± 0.000	0.800 ± 0.000
	DL	0.005 ± 0.000	0.002 ± 0.000	0.800 ± 0.000
	GS	0.050 ± 0.000	0.003 ± 0.000	2.000 ± 0.000
	DS	0.001 ± 0.000	0.004 ± 0.000	1.000 ± 0.000
100% roughage	GL	0.002 ± 0.001	0.004 ± 0.000	0.770 ± 0.001
	DL	0.002 ± 0.001	0.002 ± 0.000	0.800 ± 0.0005
	GS	0.005 ± 0.002	0.002 ± 0.000	2.000 ± 0.007
	DS	0.002 ± 0.0005	0.001 ± 0.000	1.000 ± 0.001

*GL* = Green liquid, *DL* = Dry liquid, *GS* = Green solid, *DS* = Dry solid.

**Table 5:** Comparison of Methanomicrobiales percentage obtained by qPCR and MG-RAST analysis for same samples processed by shotgun and amplicon sequencing

		**Methanomicrobiales (% ± SD)**	
**Sample**		**qPCR**	**Shotgun**
50% roughage	GL	0.05 ± 0.0002	0.08 ± 0.000
	DL	0.38 ± 0.005	0.03 ± 0.000
	GS	0.02 ± 0.008	0.13 ± 0.0005
	DS	0.01 ± 0.0002	0.23 ± 0.003
75% roughage	GL	0.20 ± 0.001	0.15 ± 0.0006
	DL	0.60 ± 0.0006	0.10 ± 0.000
	GS	0.01 ± 0.000	0.09 ± 0.000
	DS	0.002 ± 0.000	0.09 ± 0.000
100% roughage	GL	0.06 ± 0.0005	0.07 ± 0.0001
	DL	0.16 ± 0.001	0.15 ± 0.0006
	GS	0.002 ± 0.000	0.09 ± 0.0001
	DS	0.04 ± 0.0004	0.10 ± 0.000

*GL* = Green liquid, *DL* = Dry liquid, *GS* = Green solid, *DS* = Dry solid.

## Discussion

In the present study, we report the enumeration of rumen bacterial species using qPCR and its comparison with the MG-RAST based microbial profiling of rumen metagenome by shotgun and amplicon sequencing. We observed that there was no statistically significant difference between the results from molecular approach using real time PCR and results obtained by the MG-RAST based taxonomic analysis of the shotgun metagenome sequence data, except for *Prevotella*.

Molecular-based analysis using real time quantification method have revealed that cultured bacterial species represent only a small fraction of the total bacterial diversity (Janssen 2006; Stevenson and Weimer 2007). qPCR is now increasingly being used as a molecular method for enumerating bacterial load in complex microbiota like rumen (Klieve et al. 2003; Tajima et al. 2001). The enumeration by qPCR is calculated with standard curves for different bacterial species. The abundance of bacterial species is calculated as a fraction of the total ruminal bacterial population.

The evolution of high-throughput sequencing technologies and their subsequent improvements in generating reads have enabled the utilization of different bioinformatic tools for studying communities of complex flora. Access to large amounts of data gives a better account of sample diversity and at a reasonable cost. Any metagenomics data generated thus requires proper storage and analysis, one of the currently used servers for the same includes the MG-RAST. Such freely available public resources for the analysis of metagenome sequence data have surpassed the primary bottlenecks in metagenome sequence analysis like the availability of high-performance computing for annotating the data.

### Enumeration of bacterial and archaeal species

Rumen fluid samples were collected at 3 h post feeding and the cell populations were calculated for the same, since a 3 h post feed would allow sufficient cell proliferations and adherence of few species on feed particles and microbes. Total bacteria population was similar among treatments and ranged in 5.0 × 10^6^ to 6.0 × 10^7^ copies μL^-1^ rumen content. Such results were also obtained by (Pilajun and Wanapat 2012; Vinh et al. 2011).

*Prevotella* species have been observed to be dominant in rumen suggesting their importance in metabolism of dietary feed (Bekele et al. 2010; Wood et al. 1998). Among them *P. ruminocola* and *P. bryantii* are representative species of sugar fermenting microbes in the rumen. Our results also showed the dominance of these species, in particular *P. bryantii* in the liquid fraction of the rumen samples as observed by both, qPCR and shotgun sequencing based taxonomic analysis. *P. bryantii* is a gram-negative bacterium that utilizes the soluble polysaccharides, namely xylans justifying its higher abundance in the liquid fraction as compared to the solid fractions of rumen samples. This type of activity, polysaccharide utilization, plays an important role in most complex gut niches.

Cellulolytic bacteria *Fibrobacter succinogens* and *Ruminoccocus flavefaciens* showed greater abundance than that of other species of bacteria like the *Selenomonas ruminantium.* High prevalence of cellulolytic bacteria of the genus *Fibrobacter* and *Ruminoccocus* is representative of its already reported importance as the major cellulolytic species in the rumen ecosystem. These species are found to play major role in improving the fiber metabolizing ability of rumen (Shi and Weimer 1996). The population of *F. succinogens* was higher as compared to *R. flavefaciens* across the feeding treatments for majority of samples. Such results were also observed earlier for the abundance of cellulolytic species in the rumen of buffalo (Wanapat and Cherdthong 2009).

In annotation, similarity search based on the prediction of 16S rRNA gene is found to be convenient due to highly conserved gene sequences but similar to results observed by us; earlier reports have also shown that the annotations based on ribosomal genes are often found to be ambiguous (Lin et al. 2008). Few reasons for such ambiguity include the impact of partial sequences in databases due to the difficulty in covering both ends of the gene with universal primers commonly used for determining 16S rRNA gene sequences. Such sequences compete with complete rRNA sequences in sequence similarity searches. The anti Shine Dalgarno sequence, one of the most conserved features in 16S rRNA genes is evidently critical to translation efficiency (Lin et al. 2008; McCarthy and Brimacombe 1994). Anti-SD sequence being located at the 3′ end, it is likely to be excluded from 16S rDNAs obtained using molecular approaches. In their study of 16S rRNA genes, (Lin et al. 2008) observed that at least 67 out of 252 different species of the complete microbial genomes were annotated inaccurately and the main cause for such annotation was found to be the 16S rRNA partial sequences.

*S. ruminantium*, gram negative grows on substrates like fructose, glucose, mannose, cellobiose, maltose, sucrose, and salicin, producing end products from glucose like lactate, acetate, propionate, and formate. On the basis of the sugar fermentation characteristics, *Selenomonas ruminantium* has been reported to be one of the major species of *Selenomonas* genus. Similar results were also predicted by MG-RAST analysis which showed > 90% abundance of *Selenomonas* genus in all samples at all concentrations.

Ruminal methane is formed by the action of methanogens present in ruminants and are capable of using H_2_/CO_2_; can multiply easily and are observed in high abundance in rumen (Zinder 1993). Contradictorily, in our results, methanogenic microbiales were found in lesser proportion. The probable reason could be their prevalence in a symbiotic interaction with protozoa. Protozoa population will be low at 3 h post fed sampling time thereby subsequently reducing the proportion of methanomicrobiales (Sahoo et al. 2000). Dilution of the feed particles at 3 h post feed might have an impact on the growth of rumen methanogens as they are slow growers.

The present study reveals reliability of database based taxonomic profiling of rumen metagenome by validating through the already established molecular approach like the qPCR. As observed from the one way ANOVA analysis, there is concordance between the qPCR and shotgun sequencing whereas the qPCR and amplicon sequencing showed significant variation. The results are further supported by the Spearman’s rank correlation test that shows significant positive correlation between the qPCR and shotgun sequencing. Such techniques can also be applied to check the abundance of bacterial species in other complex microbial communities. Results reflect the ability of the microbes to adapt to the conditions and compete for the nutrients available; further signifying its importance to the overall functions of the complex community. Knowledge about the proportion of ruminal microbes inhabiting the rumen of a particular animal could also be used to manipulate the feeding regimes for ruminants.

## Competing interests

The authors declare that they have no competing interests.

## Supplementary Material

Additional file 1: Online resource 1 Overview of the pipeline used for animal experiments and further evaluation of bacterial populations in the complex rumen metagenomic community. Click here for file

Additional file 2: Online resource 2 Total rumen bacterial population enumerated by real time PCR. Click here for file

Additional file 3: Online resource 3 Comparison of *Fibrobacter succinogens* percentage by qPCR and MG-RAST analysis based on ribosomal RNA genes. Click here for file

Additional file 4: Online resource 4 Comparison of *R. flavefaciens* percentage by qPCR and MG-RAST analysis based on ribosomal RNA genes. Click here for file
