# Supplementary material for: Comparative evaluation of rumen metagenome community using qPCR and MG-RAST
Source: AMB Express. 2013 Sep 11;3:55. doi: 10.1186/2191-0855-3-55 (PMC3851495; doi:10.1186/2191-0855-3-55)
Supplement: Additional file 1: Online resource 1 — Overview of the pipeline used for animal experiments and further evaluation of bacterial populations in the complex rumen metagenomic community. [file 2191-0855-3-55-S1.doc]

**Comparative evaluation of rumen metagenome community using qPCR and MG-RAST**

**Neelam M. Nathani^1^, Amrutlal K. Patel^1^, Prakash S. Dhamannapatil^1^, Ramesh K. Kothari^2^, Krishna M. Singh^1^ and Chaitanya G. Joshi^1^**

**^1^**Department of Animal Biotechnology, College of Veterinary Science & Animal Husbandry, Anand Agricultural University, Anand-388 001, Gujarat, India

^2^Department of Microbiology, Christ College, Vidhya Niketan, P.B. No.05, Rajkot-5, Gujarat, India

**Correspondence:**

Dr. C. G. Joshi,

Professor, Department of Animal Biotechnology

College of Veterinary Science & Animal Husbandry

Anand Agricultural University,

Anand-388 001, Gujarat, India

**Email-** [cgjoshi@rediffmail.com](mailto:cgjoshi@rediffmail.com)

**Phone –** +91 2692 261201

**Fax -** +91 2692 261486

**Online resource 1.** Overview of the pipeline used for animal experiments and further evaluation of bacterial populations in the complex rumen metagenomic community

8 Animals (Mehsani breed of buffalo)

4 Animals **GREEN** roughage 4 Animals **DRY** roughage

Dietary Feed Regimes (Each of 6 weeks)

50% Roughage : 50% Concentrate

75% Roughage : 25% Concentrate

100% Roughage

Rumen fluid collection

LIQUID Fraction SOLID Fraction

8 Animals x 3 Regimes = 24 x 2 fractions = 48 samples

**Sequencing qPCR**

**(Shotgun and Amplicon methods)**

4 Bacterial species + Methanomicrobiales

Analysis using MG-RAST Enumeration as compared to total

(Shotgun Sequenced & Amplicon sequenced) bacterial population

Comparative Evaluation and Statistical Analysis
