# Supplementary material for: Comparative evaluation of rumen metagenome community using qPCR and MG-RAST
Source: AMB Express. 2013 Sep 11;3:55. doi: 10.1186/2191-0855-3-55 (PMC3851495; doi:10.1186/2191-0855-3-55)
Supplement: Additional file 2: Online resource 2 — Total rumen bacterial population enumerated by real time PCR. [file 2191-0855-3-55-S2.doc]

**Comparative evaluation of rumen metagenome community using qPCR and MG-RAST**

**Neelam M. Nathani^1^, Amrutlal K. Patel^1^, Prakash S. Dhamannapatil^1^, Ramesh K. Kothari^2^, Krishna M. Singh^1^ and Chaitanya G. Joshi^1^**

**^1^**Department of Animal Biotechnology, College of Veterinary Science & Animal Husbandry, Anand Agricultural University, Anand-388 001, Gujarat, India

^2^Department of Microbiology, Christ College, Vidhya Niketan, P.B. No.05, Rajkot-5, Gujarat, India

**Correspondence:**

Dr. C. G. Joshi,

Professor, Department of Animal Biotechnology

College of Veterinary Science & Animal Husbandry

Anand Agricultural University,

Anand-388 001, Gujarat, India

**Email-** [cgjoshi@rediffmail.com](mailto:cgjoshi@rediffmail.com)

**Phone –** +91 2692 261201

**Fax -** +91 2692 261486

**Online resource 2.** Total rumen bacterial population enumerated by real time PCR

| **Sample** | | **Total bacteria (ng/µL)** |
| --- | --- | --- |
| **50% roughage** | GL | 2.9 * 10^7^ |
|  | DL | 2.6 * 10^7^ |
|  | GS | 2.1 * 10^7^ |
|  | DS | 6.0 * 10^7^ |
| **75% roughage** | GL | 1.2 * 10^7^ |
|  | DL | 1.3 * 10^7^ |
|  | GS | 2.4 * 10^7^ |
|  | DS | 8.0 * 10^6^ |
| **100% roughage** | GL | 5.0 * 10^6^ |
|  | DL | 2.4 * 10^7^ |
|  | GS | 1.5 * 10^7^ |
|  | DS | 2.8 * 10^7^ |

*GL= Green liquid, DL= Dry liquid, GS= Green solid, DS= Dry solid*
