# Supplementary material for: Comparative evaluation of rumen metagenome community using qPCR and MG-RAST
Source: AMB Express. 2013 Sep 11;3:55. doi: 10.1186/2191-0855-3-55 (PMC3851495; doi:10.1186/2191-0855-3-55)
Supplement: Additional file 3: Online resource 3 — Comparison of Fibrobacter succinogens percentage by qPCR and MG-RAST analysis based on ribosomal RNA genes. [file 2191-0855-3-55-S3.doc]

**Comparative evaluation of rumen metagenome community using qPCR and MG-RAST**

**Neelam M. Nathani^1^, Amrutlal K. Patel^1^, Prakash S. Dhamannapatil^1^, Ramesh K. Kothari^2^, Krishna M. Singh^1^ and Chaitanya G. Joshi^1^**

**^1^**Department of Animal Biotechnology, College of Veterinary Science & Animal Husbandry, Anand Agricultural University, Anand-388 001, Gujarat, India

^2^Department of Microbiology, Christ College, Vidhya Niketan, P.B. No.05, Rajkot-5, Gujarat, India

**Correspondence:**

Dr. C. G. Joshi,

Professor, Department of Animal Biotechnology

College of Veterinary Science & Animal Husbandry

Anand Agricultural University,

Anand-388 001, Gujarat, India

**Email-** [cgjoshi@rediffmail.com](mailto:cgjoshi@rediffmail.com)

**Phone –** +91 2692 261201

**Fax -** +91 2692 261486

**Online resource 3.** Comparison of *Fibrobacter succinogens* percentage by qPCR and MG-RAST analysis based on ribosomal RNA genes

| **Bacterial Sample species** | | ***F. succinogens* (% ± SD)** | |
| --- | --- | --- | --- |
|  |  | **qPCR** | **MG-RAST: RNA** |
| **50% roughage** | **GL** | 0.33 ± 0.005 | 1.69 ± 0.009 |
|  | **DL** | 0.35 ± 0.001 | 2.50 ± 0.014 |
|  | **GS** | 0.20 ± 0.001 | - |
|  | **DS** | 0.11 ± 0.0007 | 0.28 ± 0.002 |
| **75% roughage** | **GL** | 0.40 ± 0.003 | 1.02 ± 0.001 |
|  | **DL** | 0.38 ± 0.003 | 1.49 ± 0.010 |
|  | **GS** | 0.10 ± 0.0004 | 0.29 ± 0.001 |
|  | **DS** | 0.02 ± 0.000 | 0.48 ± 0.001 |
| **100% roughage** | **GL** | 0.20 ± 0.002 | 0.61 ± 0.002 |
|  | **DL** | 0.78 ± 0.003 | 1.20 ± 0.001 |
|  | **GS** | 0.06 ± 0.0005 | 0.18 ± 0.001 |
|  | **DS** | 0.01 ± 0.000 | 0.23 ± 0.001 |

*GL= Green liquid, DL= Dry liquid, GS= Green solid, DS= Dry solid*
